# A Survey of the Practice and Perspectives of Chinese Acupuncturists on *Deqi*

**DOI:** 10.1155/2015/684708

**Published:** 2015-04-15

**Authors:** Yu-lan Ren, Tai-pin Guo, Huai-bin Du, Hua-bin Zheng, Ting-ting Ma, Li Fang, Yu-jie Gao, Xu-guang Yang, Xue-zhi Li, Jing Shi, Liang Chen, Yi-wei Liu, Ru-wen Zhang, Hui Zheng, De-hua Li, Xi Wu, Fan-rong Liang

**Affiliations:** ^1^School of Acupuncture Moxibustion and Tuina, Chengdu University of Traditional Chinese Medicine, Chengdu 610075, China; ^2^The Affiliated Hospital, Chengdu University of Traditional Chinese Medicine, Chengdu 610075, China; ^3^The Third Affiliated Hospital, Zhejiang University of Traditional Chinese Medicine, Hangzhou 310005, China; ^4^School of Traditional Chinese Medicine, Ningxia Medical University, Yinchuan 750004, China; ^5^School of Acupuncture Moxibustion and Tuina, Henan University of Traditional Chinese Medicine, Zhengzhou 450008, China; ^6^School of Traditional Chinese Medicine, Chongqing Medical University, Chongqing 401331, China; ^7^Yunnan Province Hospital of Traditional Chinese Medicine, Kunming 650021, China

## Abstract

*Deqi* refers to the special sensation and reaction sensed mainly by both acupuncturist and patient when a needle was inserted into the acupoints and is considered to be vital to achieve acupuncture effect. For acupuncturist, it is important to judge and control *Deqi* in clinical practice. However, enough attention is paid to patients' feelings rather than acupuncturists' nowadays. We thus conducted this survey to determine acupuncturists' perspectives about *Deqi* and to further find the proper way to induce *Deqi*. A total of 250 questionnaires were sent out to acupuncturists and 202 (80.8%) were returned. According to the results, most acupuncturists believe that *Deqi* is vital to obtain preferable clinical effects. The reliability of acupuncturists' *Deqi* sensation ranks as sinking> tightening> astringent. The reliability of patients' *Deqi* sensations ranks as sourness> numbness> distention> heaviness> pain. The reliability of influential factors ranks as manipulation> specificity of acupoint> TCM constitution> disease status> patient's psychological condition> acupuncturists' psychological guidance> clinical environment. This study is believed to provide additional evidence to the qualitative and quantitative research of *Deqi* in the future.

## 1. Introduction

Acupuncture is one of the major treatment modalities in traditional Chinese medicine (TCM). For more than 2500 years of practice, it has been widely accepted by general population in China for its curative effect, wide range of indication, simplicity, and safety in practice. It has gradually become a global therapeutic method in recent decades. According to the theory of traditional acupuncture, the effect of acupuncture is achieved by regulating the channel* Qi*. Therefore,* Qi *arrival (*Deqi*), also known as needling sensation, is considered to be closely related to the acupuncture efficacy [1]. In clinical practice,* Deqi *is measured by the sensation of acupuncturists' finger and patient's reaction. Generally speaking, when* Deqi *occurs, an acupuncturist may feel sinking (*Chen*), astringent (*Se*), and tightness (*Jin*) around the needle by his/her fingers. Meanwhile, the patient may sense soreness (*Suan*), numbness (*Ma*), distention (*Zhang*), and heaviness (*Zhong*) around the acupoint; sometimes,* Deqi* sensation could be different like coldness, warmness, pain electric-shock feeling, and so forth. However, the intensity and property of acupuncture* Deqi* may differ as a result of the different physical and psychological conditions of individuals, which made it difficult to be comprehensively applied in clinics.

*Deqi *sensation scale, as an important qualitative and quantitative measuring tool for* Deqi*, was applied to acupuncture clinical trials and mechanism studies recently [2–5]. In 1989, the Vincent* Deqi* scale was invented with 20 adjectives based on the McGill pain questionnaire. And then there were the Park* Deqi *scale and the MacPherson* Deqi *scale followed by [6, 7]. However, these scales had mainly focused on the patients' sensations but no attention had been paid to the* Deqi* sensation of acupuncturist. Further, the Southampton* Deqi *scale was drafted based on the suggestions of both patients and acupuncturists but failed to discriminate the noxious pain sensation from* Deqi* sensations according to a German trial [8, 9]. The Massachusetts general hospital acupuncture sensation scale (MASS), which was modified based on the subjective acupuncture sensation scale (SASS) [10], was composed of various needling sensations and had a measurement of the spreading of* Deqi* and patient's anxiety during needling. It has already been validated as a reliable and valid tool to measure* Deqi* in healthy young Chinese people [11]. However, till now no standardized, valid, and reliable* Deqi* scale has been formed due to the lack of sufficient evidence.

In TCM classics, the process to achieve* Deqi* is also called “*Qizhi,*” which means* Qi* arrival or* Qi* obtained through acupuncturist's manipulations after needle insertion. One of the chief indicators of achieving* Deqi* is the sensation change felt by acupuncturist's fingers. The activation, retention, and spreading of* Deqi* are closely and directly related to acupuncture manipulation techniques, while recent literature indicates that research attention has been merely paid to the patients' or healthy subjects' needle sensations, with ignorance of the* Deqi* sensation felt by acupuncturists. Therefore, we conducted this survey by consulting acupuncturists, who are engaging in clinical practice in Chinese hospitals, to explore acupuncturist's perspectives on* Deqi* and to further understand if there are different views among acupuncturists with different levels of experience.

## 2. Methods

The questionnaire for measuring acupuncture* Deqi* in acupuncturist was initially designed by 2 senior acupuncture experts, together with 2 clinical acupuncturists and 2 doctoral candidates. All participants of questionnaire design embrace rich experiences in acupuncture treatment with skillful acupuncture manipulation techniques. The current questionnaire we presented in this paper is an autonym questionnaire in Chinese that is finally completed after 5 times revisions according to acupuncture experts suggestions and comments. It contains three parts with 16 items relating to acupuncture* Deqi*. Part 1 includes 2 single choice questions and 1 open-ended question regarding the acupuncturist's thoughts on the relationship between* Deqi* and acupuncture efficacy. The questions in part 2 include 2 single choice questions, 3 multiple choice questions, and 2 questions for multiple choices, aimed to identify the proper way to judge* Deqi* in clinical practice. The questions in part 3 include 4 single choice questions, 2 multiple choice questions, and 1 question for multiple choices, aiming to find the proper manipulation for a better control and guidance of* Deqi* in clinical practice.

According to the latest literature [12], 90% acupuncturists thought* Deqi* was related to clinical efficacy. Based on assumption that it reached 96% in this study, a sample size of 200 at least is needed to achieve 90% power to detect a statistical significance by using a two-sided binomial test. The target significance level was 0.05. Thus, 250 acupuncturists were needed assuming a 25% dropout rate. A total of 250 questionnaires were sent out to 250 acupuncturists regardless of age or gender in 44 hospitals with express delivery. The included hospitals were geographically distributed in* Zhejiang, Jiangxi, Hunan, Shanxi, Qinghai, Sichuan, Yunnan, and Guizhou* province and* Beijing, Tianjin, and Chongqing* municipal city. The rationale for choosing these hospitals in different areas of different directions of China is aiming to avoid deviations caused by the dominance of a specific acupuncture theory or schools in one region. Among them, there were 34 top grade hospitals (77.2%), and most of them are TCM hospitals, 7 second grade hospitals (16%), and 3 community hospitals (6.8%). All questionnaires were completed by included acupuncturists independently.

The data of questionnaire was collected back-to-back by two researchers. Then, the data was completely and accurately transferred to computerized database for data processing including double data entry, edit checks, data cleaning, coding, and reconciliation. Continuous variables were summarized as means (SDs) and discrete data as frequency and percentage.

## 3. Results

### 3.1. Participants

250 questionnaires were sent out, and 202 (80.8%) were returned. 49.5% of the respondents were male. The participants were aged from 19 to 59 years (mean, 33.5), with working years ranging from 1 to 45 years (mean, 9.0 years) (1–5-year experience, *N* = 96; 6–10-year experience, *N* = 49; 10+ years of experience, *N* = 57). Regarding the education level, there are 8 with associate degree, 93 participants with bachelor's degree, 73 with master degree, and 18 with Ph.D. degree. Regarding technical title, there were 7 physician assistants, 98 resident doctors, 43 attending physicians, 41 associate physicians, and 13 chief physicians.

### 3.2. Perspectives on the Relationship between* Deqi* and Acupuncture Efficacy

As shown in Table 1, regarding the question “whether the* Deqi *was crucial to clinical efficacy,” 194 acupuncturists responded, and 85.57% of them thought that in most cases* Deqi *was crucial to clinical efficacy, and 8.76% thought it was absolutely crucial, while 2.06% of them stated that in most cases* Deqi *is not vital to therapy, and 3.61% of them were not sure. No one chose that* Deqi *was not related to treatment at all. Regarding the question “whether a higher intensity of* Deqi *resulted in a more preferable efficacy,” most acupuncturists (65.80%) did not think so, while 34.20% of the participants agreed. For the reason why the higher* Deqi* intensity did not yield better efficacy, some acupuncturists (45.67%) thought it was related to patients' acceptance and tolerability to acupuncture, because a higher intensity possibley resulted in harm of healthy* qi* or even noxious stimulation. 26.77% thought it was attributed to individual difference. 3.94% thought it was complicated. And 23.62% did not give any answers.

### 3.3. The Way to Judge* Deqi*

As shown in Table 2, Question (3) is a multiple choice question, and acupuncturist could make one or more choices. 87.56% of the acupuncturists said they jndged the occurrence of* Deqi* according to patient's sensation, 81.35% based on their personal sensation felt by fingers, and 36.27% according to the facial expression of patient. Question (4) demonstrated that there is only a small chance when* Deqi* was felt by acupuncturist but not by patient. The results showed that 50.52% of acupuncturists thought the probability was 5%–10%, and 26.04% thought it is less than 5%. Regarding Question (5), 88.14% of the acupuncturists could tell whether the* Deqi* was achieved just by their manipulating fingers' sensation regardless of patient's sensation.

**Figure 1:**
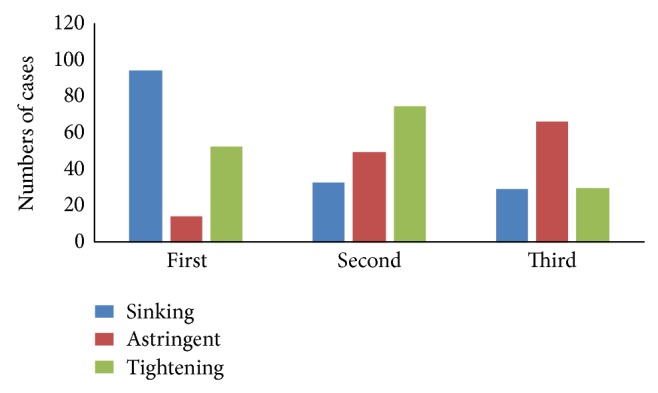
The reliability order of acupuncturists'* Deqi* sensations in Question (6).

Question (6) was also a multiple choice question. The highest frequency of any sensation felt by the acupuncturists during* Deqi* was tightening (97.30%), followed by sinking (95.14%), astringent (76.22%), and others (3.78%). In Question (7), acupuncturists ranked the reliability of telling* Deqi* according to their sensations shown in Question (6) in sequence.  Figure 1 demonstrated that acupuncturist believed that sinking was ranked at the first place, while tightening the second, and astringent the third, in the reliability of telling* Deqi* based on their personal sensations.

**Figure 2:**
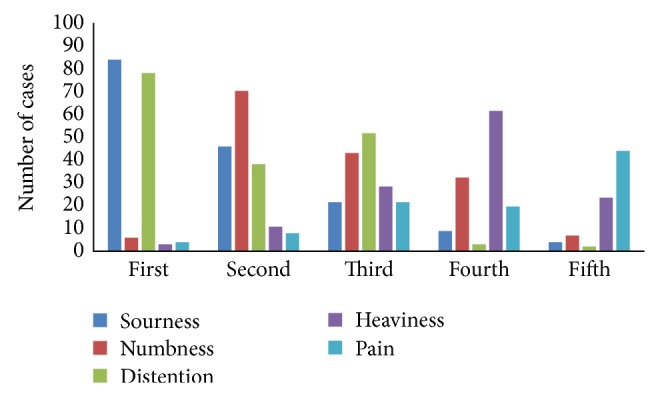
The reliability order of patients'* Deqi *sensations in Question (8).

Answers to Question (8) demonstrated that main sensations of* Deqi* reported by patients were distention (96.43%), soreness (92.86%), numbness (86.73%), heaviness (70.92%), and pain (53.06%). In order to determine the reliability of telling* Deqi* by patient's subjective sensation, acupuncturists were required to answer Question (9). The result in Figure 2 showed that acupuncturist believed that soreness was ranked at the first place, while numbness the second, and distention the third, followed by heaviness and pain, in the reliability of telling* Deqi* based on patients' subjective sensations.

### 3.4. The Way to Control* Deqi*

**Figure 3:**
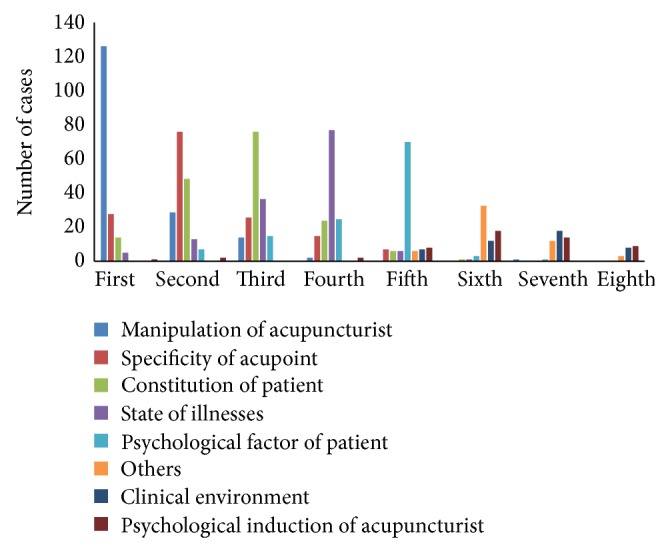
The order of influencing factors of* Deqi* in Question (16).

As shown in Table 3, Question (10), regarding the influential factors of* Deqi* during clinical practices, was a multiple choice question. The main influential factors of* Deqi *were manipulation (98.43%), patient's body constitution (95.29%), acupoint (87.43%), the state of illness (80.10%), and psychological condition of patient (68.06%). The importance and reliability of* Deqi*'s influential factors to* Deqi* was ranked in the order as manipulation of acupuncturist> specificity of acupoints> constitution of patient> status of illness> pathogenetic condition of patient, as shown in Figure 3.

Regarding Question (12), the most frequently used manipulation to promote* Deqi* sensations was the combination of rotating, lifting, and thrusting (72.54%), and supplementary manipulations such as scrape, shake, and press were also used by some acupuncturists (17.62%), while just one kind of manipulation was seldom applied. The questions (13) showed that most acupuncturists (74.21%) believed that the depth of insertion to elicit* Deqi* sensation should be based on the patient's conditions, and 25.79% thought that deep insertion was easier, but few chose shallow insertion. In response to the question of whether the strong sensation produced by rotating the needle in single direction was true* Deqi* or not, 46.87% acupuncturists agreed with this point. According to Question (15), the majorities of acupuncturists (98.45%) are able to induce* Deqi* right at the moment of needle insertion, even without needle manipulation. But the possibility was not high for 21.05% of acupuncturists thought it <20% and 38.42% of acupuncturists thought 20%–40%, indicated in Question (16).

## 4. Discussions

### 4.1. *Deqi* Is Crucial to Acupuncture Efficacy

In our study, 94.33% acupuncturists stated* Deqi *was crucial to clinical efficacy. Of these, 85.57% thought it was crucial in most cases while 8.76% thought it was always crucial. No one denied its importance completely. A recently randomized controlled trial (RCT) of Bell's palsy demonstrated that the strong* Deqi *could result in better outcomes [2], while another study stated no pain relief in osteoarthritis patients [13]. Regarding the intensity, the majority of acupuncturists did not believe that a stronger intensity would increase the benefits while 34.20% agreed. This result was similar to the results reported by Han [14], who showed that a low frequency (2 Hz) had better effects on easing pain than a high frequency (100 Hz). There is no doubt that* Deqi *is crucial to clinical efficacy in our study. However, future clinical trials are required to confirm this.

### 4.2. *Deqi *and Acupuncture Manipulations

Our investigation showed that the most significant influencing factors of* Deqi *were the acupuncturist's manipulation. Acupuncturists' manipulation was also reported to be the most important influencing factor in clinical studies. It is accepted that manipulation could induce the release of* Deqi *and promote the degree of* Deqi *or alleviate the strong* Deqi *sensation. It is also known that different types of manipulation can result in different* Deqi *sensations. For promoting or controlling* Deqi*, acupuncturists preferred to use the combined manipulations of rotating, lifting, and thrusting and also used the supplemental manipulations such as scraping and shaking. As the issue of whether the strong feeling caused by rotating with one single direction was due to* Deqi*, acupuncturists themselves did not come to an agreement. Sometimes, nearly all acupuncturists have experienced that* Deqi *occurred just as the needle was inserted without any other manipulations, but the probability was very low.

Different depth of insertion has aroused considerable debate, and more researchers favor the idea that with deep insertion it is easier to produce* Deqi *sensations. Functional magnetic resonance imaging (fMRI) has shown that deep electroacupuncture on GB34 and GB35 could generate stronger* Deqi *sensations and more effectively modulate the pain-related neuromatrix than shallow electroacupuncture [15]. Other studies also proved that deep acupuncture could result in higher* Deqi *sensations scores [16] and increase the skin and muscle blood flow [17] in healthy subjects. However, some studies report the opposite views. A fMRI research declared no significant differences in the blood oxygen level dependent (BOLD) responses by the deep and shallow stimulations [18]. RCT also showed that the same effects were achieved by both deep and superficial acupuncture in idiopathic anterior knee pain patients [19]. Our questionnaire results demonstrated that no one thought the shallow insertion made it easier to produce* Deqi *sensations, and 25.79% of the acupuncturists stated that deep insertion made it easier, while the majority of the acupuncturists (74.21%) explained that it depended on the patients' conditions and was not relevant to the depth of insertion. The shallow stimulation usually means that the needle tip reaches the subcutaneous tissue, while the deep stimulation may arrive at the muscular or nervous tissues.* Deqi *sensation may not be completely relevant to the deep tissues. One study provided evidence that* Deqi *was not relevant to the deep median nerve contact nor median nerve penetration during needling in P6 point with ultrasound measurement [20]. Another study also explained that it depended on the patients' conditions and no relevance was attributed to the depth of insertion because in healthy subjects the skin and muscle blood flow increased with no significance comparing deep and shallow stimulation, but in fibromyalgia patients there were significant differences [5].

### 4.3. The* Deqi *Sensation of Acupuncturists and Patients

It seems that the feelings of patient and acupuncturist are often used to judge whether* Deqi* has been generated or not, but few researches have demonstrated the real details of judgment. The results of this study showed that the majority of acupuncturists recognized whether the* Deqi* had been achieved just by their fingers' sensations during manipulations. These sensations were mostly tightening, sinking, and astringent, which were similar as those described in textbooks [1]. Interestingly, the* Deqi* sensations experienced by fingers were ranked as sinking, tightening, and astringent according to the reliability. In our survey, the patients' sensations were mainly described as soreness (*Suan*), numbness (*Ma*), distention (*Zhang*), heaviness (*Zhong*), and pain (*Tong*). The results showed the order of frequency as distention, soreness, numbness, heaviness, and pain, and the orders of its reliability level scores ranked as soreness>numbness>distention>heaviness>pain. For the top three sensations, although there were different words and expressions to describe Zhang, Suan, and Ma, some previous researchers also demonstrated similar results [21–24]. So,* Deqi* sensation in both acupuncturist and patient, occurrence probability, and the order of reliability were revealed. These results may provide some advice for further quantification studies for* Deqi*.

### 4.4. Influencing Factors of* Deqi*

Except for acupuncturist's manipulation, the common influencing factors of* Deqi *included the specificity of acupoint, the constitution of patient, and the patients'psychological factor as shown in our study. For the specificity of the acupoint, studies had shown that it existed and was related to clinical effects closely [25], but it needs further clinical study to determine its direct relationship with* Deqi*. Yet for all that, according to common sense, the feeling of pain is evident with needling the terminal points on the four limbs, while the soreness and distention are noted in thick muscle points. Meanwhile, the conditions of patient such as constitution, illness state, and psychology were considered as the important factors to* Deqi* in Chinese ancient and modern literatures [26–28]. As noted in “*Ling *Shu* Jing,*” the speed of* Deqi *emergence is faster in patient with* yang* excess constitution than the one with* yin* excess. Compared with healthy volunteers, patients suffering chronic pain tended to acquire a much stronger* Deqi *sensation [29]. Some researchers believe that* Deqi *is the brain awareness and consciousness because the sensations of the subjects were the same between sham laser acupuncture and true laser [30]. However, in the classical literature such as the “*Huangdi Neijing*” and the “*Zhenjiu Dacheng,*” psychological factors are important with* Deqi*, not only in the process of acupuncture but also in influencing the clinical outcomes. Clinical trials revealed that Bell's palsy patients with the personality factors of excitability, sociability, braveness, and intellectuality had an easier time to gain* Deqi *[31], and the anxiety and dominance were correlated with the treatment effects of primary dysmenorrhea [32]. In addition, the anxiety also affected the heart rate variability in healthy subjects [33].

### 4.5. Limitations

The main limitation of this survey is that the contents are designed according to Chinese acupuncturist's customs, including manipulations, acupuncturist's needling sensations, patient's sensations, and the factors of* Deqi*, and some items may be difficult to understand and do not conform with international conventions. Most of the acupuncturists came from the grade-three general province hospitals, and opinions from lower grade hospitals were insufficient. The results are from acupuncturists' general perspectives; a supplementary survey of the patients' views is necessary.

## 5. Conclusions

Our survey demonstrates that* Deqi *is important to clinical effects according to the acupuncturist's views. The integrated manipulations are the most common way to promote* Deqi *sensation. The reliability of primary acupuncturist fingers'* Deqi *sensations ranks as sinking>tightening>astringent. The reliability of primary patients'* Deqi *sensations ranks as sourness>numbness>distention>heaviness>pain as reported by patients. The reliability of primary patient's* Deqi *sensation factors ranks as manipulation>specificity of acupoint>TCM constitution>disease status>patient's psychology>acupuncturist's psychological hint>clinical environment.

In short, this paper shows the perspectives of Chinese acupuncturists on* Deqi*. The results may provide some evidences to the qualitative and quantitative research of* Deqi*. To formulate and evaluate a* Deqi* sensation scale, it may be better to include both of the sensations of acupuncturist and patient on the basis of the credible rank. In clinical research and practice, the influential factors of* Deqi *should be considered.

## Figures and Tables

**Table 1:** Perspectives on the relationship between *Deqi* and acupuncture efficacy.

Questions	Views	Respondents (*n*)/all respondents (*n*), %
(1) Do you think *Deqi* is crucial to acupuncture clinical efficacy?	① Yes, totally	17/194, 8.76%
	② Yes, mostly	166/194, 85.57%
	③ No, mostly not	4/194, 2.06%
	④ No, totally not	0
	⑤ Uncertainty	7/194, 3.61%

(2) Do you think a higher intensity of *Deqi* results in a more preferable efficacy?	① Yes	66/193, 34.20%
	② No	127/193, 65.80%

**Table 2:** The way to judge *Deqi*.

Questions	Views	Respondents (*n*)/all respondents (*n*), %
(3) Which way(s) do you prefer to judge the occurrence of *Deqi*? (multiple choices)	① According to sensation of patient's reaction.	169/193, 87.56%
	② According to sensation of your fingers	157/193,81.35%
	③ According to facial expression of patient	66/193, 36.27%

(4) What is the probability of the case that *Deqi *has been felt by your fingers but the patient reported not?	① Less than 5%	50/192, 26.04%
	② 5%–10%	97/192, 50.52%
	③ 10%–15%	26/192, 13.54%
	④ 15–20%	13/192, 6.77%
	⑤ More than 20%	6/192, 3.13%

(5) Can you recognize whether the *Deqi* has appeared just by your fingers' sensations during manipulations?	① Yes	171/194, 88.14%
	② No	23/194, 11.86%

(6) What sensations have been felt by your fingers when *Deqi* has emerged? (multiple choice)	① Sinking	176/185, 95.14%
	② Astringent	141/185, 76.22%
	③Tightening	180/185, 97.30%
	④ Others	7/185, 3.78%

(7) Please rank the acupuncturists' *Deqi* sensations (those you chose in Question (6) in the order of their reliability for telling the arrival of *Qi*.	/	Shown in Figure 1

(8) When *Deqi *occurred, what are the sensations reported by patients? (multiple choices)	① Sourness	182/196, 92.86%
	② Numbness	170/196, 86.73%
	③ Distention	189/196, 96.43%
	④ Heaviness	139/196, 70.92%
	⑤ Pain	104/196, 53.06%
	⑥ Others	7/196, 3.57%

(9) Please rank the patients' *Deqi* sensations (those you chose in Question (8)) in the order of their reliability for telling the arrival of *Qi*.	/	Shown in Figure 2

**Table 3:** The way to control *Deqi*.

Questions	Views	Respondents (*n*)/all respondents (*n*), %
(10) What is the influential factors of *Deqi* during your clinical practices? (multiple choices)	① Manipulation of acupuncturist	188/191, 98.43%
	② Specificity of acupoint	167/191, 87.43%
	③ Constitution of patient	182/191, 95.29%
	④ State of illnesses	153/191, 80.10%
	⑤ Psychological factor of patient	130/191, 68.06%
	⑥ Clinical environment	53/191, 27.75%
	⑦ Psychological induction of acupuncturist	54/191, 28.27%

(11) Please rank *Deqi*'s influential factors (those you chose in Question (10)) in order according to their reliability and importance.	/	Showed in Figure 3

(12) Which manipulation is the most commonly used to promote the arrival of *Deqi* sensations?	① Lifting and thrusting	12/193, 6.22%
	② Rotating	2/193, 1.04%
	③ Combination of rotating, lifting, and thrusting	140/193, 72.54%
	④ Supplementary manipulations like scrape, shake, and so forth.	4/193, 1.04%
	⑤ Combination of ③ and ④	34/193, 17.62%
	⑥ Others	1/193, 0.52%

(13) For deep or shallow insertion, which one more easily elicits *Deqi* sensation according to your experiences?	① Deep	49/190, 25.79%
	② Shallow	0/190, 0%
	③ Depending on patients' conditions	141/190, 74.21%

(14) For the strong sensations caused by rotating with one single direction, does this feeling belong to *Deqi*?	① Yes	90/192, 46.87%
	② No	102/192, 53.13%

(15) Have *Deqi* ever occurred as soon as the needle was inserted without any manipulations?	① Yes	190/193, 98.45%
	② No	3/193, 1.55%

(16) If (15) has occurred, what is the approximate occurrence rate?	① Less than 20%	40/190, 21.05%
	② 20%–40%	73/190, 38.42%
	③ 40%–60%	55/190, 28.95%
	④ 40–80%	19/190, 10%
	⑤ More than 80%	3/190, 1.58%
